# The Later Phases of Medical Examinations for Life Insurance

**Published:** 1887-09

**Authors:** 


### THE MEDICAL JOURNAL AND EXAMINER.

VOL. LV. SEPTEMBER, 1887. NO. 3.

### THE LATER PHASES OF MEDICAL EXAMINATIONS FOR LIFE INSURANCE.

READ AT THE ELEVENTH ANNUAL MEETING OF THE AMERICAN ACADEMY OF MEDICINE AT WASHINGTON, D. C., SEPTEMBER 3, 1887, BY C. C. BOMBAUGH, A.M. M.D., BALTIMORE, MARYLAND.

LEAVING out of consideration the groping of the earlier adventurers in the field of life contingencies, the history of American life insurance transactions dates back only forty-four years. Of this comparatively brief space, the first half was a period of inactivity; the remaining half has been signalized by a movement whose development, expansion and results are unparalleled in the annals of financial enterprise. In spite of the drawbacks of a protracted monetary panic, the discouragements of incompetent and fraudulent management, and the antagonism of spurious and counterfeit forms of insurance, twenty-six leading American companies have, within the past twenty years, paid obligations to the extent of one thousand millions of dollars; they hold accumulated available funds to the amount of five hundred and sixty millions of dollars; and they have incurred steadily maturing liabilities to the amount of two thousand two hundred and fifty millions.

Like the temple of Gaza, the structure of life insurance has two chief supporting pillars. The one is the mathematical, the other is the medical. The one we leave to the actuary; be it our care to see that no mischievous or antagonistic forces are applied to the other. The worshipers of Dagon who were crushed by the falling roof of the Philistine temple paid with their lives the penalty of the scoffs and insults and mockeries which they heaped upon the Israelitish Hercules. The fall of the temple with which we are so directly concerned, and under whose roof is sheltered the prudential provision and the expectant support of multitudes of American households, would be infinitely more disastrous because it would be a blow to thrift and saving.

During the introductory period of the life business in this country the blanks furnished by the companies for the medical examiners were characterized by simplicity and conciseness. These simple forms, with occasional additions in the way of greater breadth or comprehensiveness in the grouping or classification of the questions, were retained during a considerable portion of the high pressure period. Anxiety to build up the business and the sharp stimulus of competition moderated the rigidity of medical scrutiny. Of course, the range of disqualification was as clearly defined and rational grounds of rejection were as restraining and controlling as they are to-day. The approval by the executive management of mild measures in the medical department did not imply neglect of the safeguards. Moreover, the absence of cast-iron inflexibility in medical selection was counter-balanced by the stringent restrictions of the contract, voiding the policy in case of misrepresentation, concealment or suppression of facts material to the risk, suicide whether sane or insane, duelling, death by the hand of justice, extra-hazardous occupation, intemperate habits, and travel or residence in unhealthy climates.

These stipulations led to frequent misunderstanding, disappointment and litigation. It was perceived that the con tract was a one-sided covenant, that its conditions were oppressive, that within its framework were means and appliances of invalidation, and that technical pleas in avoidance of settlement of claims were sustained by rulings of the courts. Thoughtful men became impatient over repeated contests on the part of litigious managers, and declined to enter into agreements which involved hardships never contemplated. They did not choose to leave to their families a legacy of prospective litigation, and the life business suffered accordingly.

Then followed the peace-offering of non-forfeiture, and the still larger concession of incontestability. Under a a broader policy, and a more liberal line of action, the business in the hands of irrepressible agents revived. By these signs they were enabled to conquer. Non-forfeiture plans and methods, whether enforced by statute law or voluntarily adopted by the companies, derived their acceptability from their inherent fairness and their manifest equity. Sacrifices from lapse and legal default were exchanged for the recompense of even-handed justice. But the incontestability clause within the last seven or eight years has gone farther. It is not merely just, but generous. It sweeps the old ironclad restrictions off the face of the policy. The introduction of the feature of indisputability places the policy under a statute of limitations. It is a guarantee of protection against the technicalities with which the old policies were loaded, and the complications with which they were befogged. What is the practical effect of this surrender of the old conservatism, this radical concession which makes inalienable what was formerly held upon uncertain tenure? Obviously, it is encroachment upon the domain of the moral hazard. Whence, then, is to come the balance, the compensation, the counterpoise? The question receives its answer in a requisition upon the medical department of the service to give increased attention to the physical hazard.

The range of investigation, both as to the family record, and the individual history and condition, is largely extended. Simplification gives way to amplification; the more searching questions are reiterated; divisions are subdivided. While some companies, however, even in this process of closer scrutiny, confine inquiry to essentials, others insist upon the temperature as well as the pulse, upon spinal measurements as well as thoracic expansions, upon unvarnished reports of habits as to stimulants and narcotics as well as hereditary transmission, upon urinary tests in all cases instead of limiting analysis to ages over forty, and amount applied for over $5,000. Others go still farther in widening the scope of the examiner's duty and responsibility. They require him to fill blanks formerly filled by the agent, thereby placing him in a new relation to the company, the agent, and the applicant. He is not merely a sentinel on the watch tower, but he becomes a virtual detective. He puts the applicant on his good behavior as to future disqualification, and extorts from him an agreement, in case of eventual hernia, to wear a suitable truss. His judgment is invoked as to the insurance elements of a risk as well as to the points of medical selection. If the applicant is not up to the gilt-edged standard of acceptability, and under the rules of the company not eligible for ordinary life policies, and only eligible for term policies or short endowments, he becomes the arbiter, not only in the measurement of the expectation of life, but, according to his estimate of probability, he determines and adjusts the form of policy. Thus, "from an advisory, he is thrust into an executive office, from a simple refereeship as to the character of a company's risks, he is put forward, in some instances, as one of the corporate representatives in the making of contracts, a duty foreign to his education and usual experience."*

Whatever may be said, one way or another, of this sort of refinement in the gradation of viability, it may be urged in favor of such effort toward precision in medical appraisement that it is the only present barrier in the way of sweeping exclusion. It provides a ticket of admission to many who would otherwise be stopped at the gateway. It softens much of the rigor and corrects much of the injustice of rejection. The advocates of that rigor, and hence the direct or indirect defenders of the injustice which proceeds from it, ignore the fact that life insurance is, or ought to be, for average lives; that its advantages should be extended in every practicable way, and not coldly refused to all but those within the charmed circle of physical perfection. If average safety is declined, if a fair and reasonable degree of insurability cannot be brought within the requirements, the life insurance system falls short of its mission, and thousands who are entitled to its protection are driven to the temporary care of ephemeral assessment associations. Therefore it is that the gradation referred to does not go far enough. We must look to compliance with the demands of simple justice and common fairness for such modification of the terms of admission as will extend the benefit of insurance to the larger constituency entitled to it. But intrusion upon the strongholds of conservatism does not imply weakening of the safeguards. To maintain the conditions of security, it is only needful to institute trustworthy means and methods of gradation—to make life risks gradable and ratable as other risks are. Establish a scale of deviations from the fixed standard, and regulate the terms of acceptance accordingly. Let the counterbalance be provided through an equitable increase of premium, or an equitable reduction of dividend; through the accommodating features of term policies, or contraction of the amount of insurance; in brief, through any mode of apportionment or equalization which growth in special knowledge and sagacity may suggest. In the field thus opened, professional skill may find a way to higher function and larger achievement, and professional pride find stronger reinforcement, than in the work of rigorous exclusion.

In this direction will also be found the best offset to facts of experience that are well calculated to tone down professional pride. The most self-opinionated hobby-rider cannot lose sight of the fact that the value of medical selection is limited to a period of less than five years. He cannot forget the

"chances strange,
Disastrous accidents, and change,
That come to all;
Even in the most exalted state,
Relentless sweeps the stroke of fate;
The strongest fall."

He cannot forget how often the athlete, with his massive frame and giant strength and armor plating, defeats his early promise through reckless abuse of his physical gifts, and falls by the wayside at an age when he should rejoice in the ripened fullness of power. He cannot help noting that while robust vigor thus exhausts its nervous energy and drops into a premature grave, the weaklings, whose application for life insurance would be regarded as a farce, cautiously avoid friction and strain and exposure, husband their impoverished vital force with studious economy, and defer the services of the undertaker till long after they pass their three score and ten. He cannot avoid seeing that the ambition to keep the mortality rates of actual experience far below the estimated ratio of the tables is by no means as laudable—so long as the premium rates are not reduced correspondingly—as it would be to approximate more closely the tabular prediction, and thus widen the area of practical beneficence. He sees

* Vice-President John M. Taylor, Conn. Mut. Life.

that the red-tape repressiveness of officialism makes insurance more expensive to the risks which are accepted, because they have to pay more to keep others out than they would lose by admitting them. He cannot escape the lesson taught by the actuary that the law of average is the law of large numbers, and that the larger the number of individual cases, the less will be the fluctuations of mortality. Nor can he forget, even in the atmosphere of bureaucracy, that while the executive officers may be men of cold blood and cast iron, men to whom business is all in all and sentiment is nothing, he himself has been trained in a liberal profession, which, in proportion to its means, devotes more time and energy to philanthropic ends than all other professions combined, and that to the extent to which he allows himself to be an instrumentality in pushing the requirements of acceptability to extremes, he is untrue to the immemorial traditions and prescripts of his craft. At the bedside, or at the operating table, he may say with Hamlet, "I must be cruel only to be kind." In dealing with absolute uninsurability, he is cruel only to be just; he is governed by what Burke calls "the cold neutrality of abstract justice." But justice may be left to assert itself and to stand on its own merits. What is attracting increasing attention to the work of the examiners is the *injustice* with which much of that work is chargeable.

This has been particularly noticeable with reference to presumed renal and cardiac disturbances. In case of detection of albumen in the urine, or of a heart murmur, functional or organic, it has heretofore been the usual practice to turn down the application without further inquiry and without regard to true diagnostic significance. The simple revelation of such unfavorable indications sufficed to evoke unfavorable prognosis. They were cautionary signals that betoken danger, and it was not considered necessary to draw lines of nice distinction as to the nature and extent of the impending danger. It was taken for granted that pathological change was in progress, the possibility of an exaggerated physiological condition not being entertained. If there was hesitation or doubt, it was speedily settled by the axiomatic clincher that the company must have the benefit of the doubt. Thousands who were thus rejected years ago are still playing their allotted parts on the great stage of the world's action.

With our improved and constantly improving means of differential diagnosis there is no warrant for attaching disproportionate importance to the appearance of albumen or sugar, or for regarding the presence of either as a fatal symptom. With our advances in medical chemistry, and the corroborative aid of the microscope, it becomes comparatively easy to distinguish the temporary from the permanent forms of albuminuria. Of course it is only by frequent and systematic testing that we determine functional or physiological albuminuria, even in the intermittent or cyclic forms, from the conditions which point to acute or chronic nephritis. But it is due to the candidate for insurance that the analysis be repeated at varying periods and under changing circumstances, until it is decisively shown whether the albumen is transient, or whether there is reason to suspect future mischief. If, at any time, the test tube is fallacious, or its testimony is negative, the microscope will supplement as a corrective. To reject a man for life insurance, and to sentence him to death from Bright's disease on one test tube examination, and without looking for and finding epithelial casts, or other renal derivatives, is absurdly unfair and unreasonable, Let it be remembered, too, that cases of chronic albuminuria have been reported without albuminous urine,* while such constant accompaniments as the typical pallor, and puffiness, the tube casts, progressive emaciation, anasarca, and uræmia were emphasized.

Nor does saccharine urine necessarily imply the approach of fatal glycosuria. Without stopping to consider whether the presence of sugar indicates anything more than a transitory consequence of conditions, physiological or pathological, which have little or no affinity to those

*Journal of the American Medical Association, June 4, 1887.

of diabetes, our examiners have been declining application after application, year after year, on the assumption of the infallibility of the copper test, and paying no attention to the absence of the rest of the clinical group which we associate with diabetes. They have been betrayed by a single misleading observation into summary rejection of intending insurers, and thereby have wrought hardship none the less oppressive because unintentional—hardship which in many cases might have been avoided by greater precaution, by repeated analysis, and by definite decision whether, aside from the presence of glucose, there were other pronounced symptoms.

No sooner was attention drawn to this too frequent rejection in consequence of the adverse result of the employment of Fehling's or Trommer's test, than it became evident that the accuracy of these favorite tests had been overrated. It was discovered that other substances as well as sugar, have the property of reducing the cupric to the cuprous oxide, and that, in proportion to their appearance and interference, the copper test is untrustworthy. Not only does an excess of urates of the protein compounds exhibit a similar property, but several medicinal substances, when taken into the system, notably chloral hydrate, chloroform, turpentine, and benzoic and salicylic acids, will occasion similar reduction if the test is made in the usual manner, by heating the urine.

Then followed the discovery by Dr. Donaldson, of Baltimore, of an unknown substance, the reaction of which, under Fehling's test, had led to the repeated rejection of a man in vigorous health upon the assumption that it was glucose. After exhaustive analyses by Profs. Tyson, Wormley and Marshall, it was found to be an acid new to medical chemistry, and which had never before been detected. Dr. Brune, of Baltimore, also showed that while in the case referred to the copper tests gave reactions similar to sugar, the fermentation test and the polariscope proved the absence of glucose. His investigations showed further that while the positive evidence not only of the copper sulphate, but likewise of the sodic hydrate test, and perhaps also the picric acid and bismuth subnitrate, is not to be relied upon, we can safely depend upon the polariscope and the yeast test, and a proper interpretation of other recognized tests for glucose. To this list, it may be observed, has recently been added the valuable phenyl-hydrazin chloride of Fischer and Ultzmann. Its success rests upon reactions and methods different from those in use heretofore, and free from the objections to color and reduction tests.

With reference to the habit of giving an unfavorable prognosis in cardiac affections, a very marked change of opinion has been in progress since the important discussion upon chronic valvular disease of the heart, at the last annual meeting of the British Medical Association. Those who took note of the proceedings will remember that the subject was introduced by Sir Andrew Clarke in an elaborate paper, with a tabular report of 684 cases of chronic valvular disease, "the presence of which was not indicated by symptoms, and did not sensibly interfere with health." The eminent gentlemen who participated in the discussion which followed fully agreed upon the following conclusions: that there has been too strong a tendency heretofore to overestimate the importance of cardiac murmurs, and to take too grave and sombre a view of such murmurs, especially when loud and obtrusive; that the time has come when the conditions of valvular disease can be so grouped that the examiner is in position to accurately estimate the degree of damage, and to equitably apportion the the extra rating to the extra risk; that mitral lesion give us more hope of amelioration through the adaptive processes of nature than aortic defects; that whatever the valvular lesion, more importance is to be attached to signs of weakness or enlargement of the heart, to displacement, to evidence of degenerative change in its walls or in the arteries, to obstruction in the circulation, and to liability to rheumatic affections. With regard to disease of the mitral valve, Sir Andrew Clarke presents five propositions: First—that there shall be no degeneration of the heart; second—that the disease shall have existed two years; third—that the ventricles shall be in good order; fourth—that the arteries shall be sound; fifth—that there is no persistent basic nor recurrent hepatic congestion; that colds shall not hang about the patient, and that his general health shall be good; given all these, and the mitral disease will not shorten life.

As to rating up the extra risk to the higher age at which the premium is more nearly adjusted to the point of adequacy, the experience of English offices has been too limited to furnish well defined guidance. The only suggestive results which have been collated and published, thus far, are those of Prof. E. Symos Thompson, who has shown very active interest in this special line of investigation.

Dr. Barlow, of London, in reviewing the interesting observations of Prof. Thompson, says:

"The whole summary of the matter seems to be that the selection and admission of these cases is one of great delicacy, and requires the highest qualifications of experience and ripe, sound judgment on the part of the physician; that though in the hands of a few men of exceptional experience, a few (very few) cases of aortic valvular disease may be admitted at a greatly advanced premium rate, yet that as a general rule for the majority of examiners the safe course will be rejection. But as to mitral disease of some standing, dating from early life, and occurring in otherwise healthy, temperate persons, subject to no laborious exertion or great anxieties, and having no enlargement or displacement of the organ, and no fatty or other degeneration of heart or arteries, an addition varying with circumstances from ten to twenty-five years should be made to the actual age."

